# Progressive Pharmacy

**Published:** 1877-08-20

**Authors:** Wm. A. Greene

**Affiliations:** Macon, Ga.


### SOUTHERN MEDICAL RECORD.

VOL. VII. ATLANTA, GA., AUGUST 20, 1877. NO. 8.

THOMAS S. POWELL, M.D.,

W. T. GOLDSTI, M. D.,

R. C. WORD, M. D., } EDITORS.

R. C. WORD, Business Manager.

SUBSCRIPTION: TWO DOLLARS PER ANNUM, IN ADVANCE.

☞ All communications, and letters on business connected with THE RECORD for 1877 must be addressed to the Business Manager.

### Original and Selected Articles.

### PROGRESSIVE PHARMACY.

#### SUGAR COATED PILLS OF THE DAY.

BY WM. A. GREENE, M. D., MACON, GA.

Those who have read the published proceedings of the American Pharmaceutical Association for the past year or two, will remember the discussions concerning "ready made pills," and in the "Journal of Pharmacy" Prof. Jos. P. Remmington and Mr. Samuel Campbell, of Philadelphia, have indulged in quite a *pill battle*, which was much safer than *bullets* though the *missiles* are so *similar*. But this is an important subject, and physicians should take care they are "posted," that their selections of these pills may be both judicious and intelligent.

For their information, I propose, in my feeble way, to briefly give my observation and experience concerning these Sugar Coated Pills, which are in such universal demand and use.

The well-known, standard ready made pills are embraced in the following list, viz: The officinal ready made pills; the "soluble pills," pills from Messrs. Schieffelin & Co., of New York; the gelatine coated pills from Keasbey & Mattison, of Philadelphia, and from McKesson & Robbins, of New York; the compressed pills from John Wyeth & Bro., and Jacob Dunton, of Philadelphia; the medicated globules or pearls from E. Fougera & Co., of New York; also the *cachet de pain*, a French wafer recently introduced—and the *Sugar Coated Pills* from *many* well-known houses. Each of these claim superior merit and value, because of the relative solubility of the officinally prepared pills, and those prepared by *compression*, or by coating with gelatine, sugar, etc. In this paper I shall particularly discuss the *sugar* coated pills of the day—for the reason above mentioned, that they are most generally manufactured and prescribed, and called for by the people. For a pill to be valuable and reliable, it must be composed of pure drugs, be equally distributed in the mass, of uniform weight, and readily and rapidly soluble in the stomach. It is a question of some difference of opinion, among those who have examined the subject, which of the above mentioned pills are most soluble, admitting they are all honestly and skilfully prepared. Unfortunately, there is great adulteration and swindling in these covered or coated pills—the temptation being so great to make money by thus covering over and hiding the impure and worthless drugs—and obtain patronage over the head of their *honest* competitors, by selling *cheap* pills. I cannot lose this opportunity to caution against *cheap sugar coated pills*—and advise the *greatest* care in mentioning the *manufacturers name*, when prescribing such pills. We cannot be too careful, especially when using *quinine* pills, as so often life depends on the prompt action of this invaluable medicine in our malarial diseases. It is admitted by all, that the officinal pill is the most soluble, provided, the proper excipient is employed in preparing them, as licorice, or what is much better, pure glycerine. Next comes the sugar coated and compressed pills, each advocated by intelligent pharmacists, as being the most soluble. In my experiments I have found very trifling difference between these two, when the sugar coated pill was properly selected—the *manufacturer*, being an important consideration.

In a trial with *nine* different manufacturers, I have found *none superior* to Bullock & Crenshaw's of Philadelphia —who nearly twenty years ago introduced sugar coated pills to the profession in the United States. When they introduced these pills there was not a firm in Philadelphia, or the state of Penn–sylvania, making them, and but one other on the Continent. Through all these long years these pills have been in the hands of all druggists, all over the land, and not a breath of suspicion has ever been whispered against their purity and reliability. No greater commendation could be asked for. These pills received, among others, the award of a Centennial medal, for superiority of finish and purity of ingredients, after a critical examination by medical men of ability and skill.

Undoubtedly a fair test of solubility would be dependent upon the varied conditions of the fluids or contents of the stomach, which cannot be obtained. We are, therefore, compelled to select a fluid as nearest approximating the average state of the dissolving powers of the organ, with a temperature of about 98° Fahrenheit, the acidity, alkalinity and digestive powers in average proportions.

After testing the solubility of all the ready-made pills before mentioned, I found from the samples furnished me, the sugar-coated pills most soluble, with conditions as above. Those used in the experiment were from those of Bullock & Crenshaw, W. R. Warner & Co., Hance Bros. & White, Reed & Carnrick, and four or five others. There was really no material difference in the sugar-coated pills of the firms named—a small advantage in favor of the first one. I will give from the tabulated record I have preserved, the result of the Bullock & Crenshaw pill, since they excelled the others.

I will take the two-grain quinine pill (sugar coated) and the Pil Cath. Co. U. S. P., as samples, (also sugar coated). In a one and a half ounce solution of water at 98 deg., acidulated—the quinine pill-coating came off in five minutes, and disintegrated in twenty minutes.

The *Pil Cath. Co., U. S. P.*, was *fully* disintegrated in *forty* minutes.

In acidulated water 98° F., and small addition of pure pepsin (E. Shiefer) quinine pill was dissolved in twenty-six minutes.

The Pil Cath. Co. U. S. P., in a state of solution in twenty–two minutes.

In each experiment the vessel containing the pills and solution was kept in constant to-and-fro motion.

Other sugar coated pills of Bullock & Crenshaw yielded relatively the same proportional results, tested with similar solutions—having in my possession the following, viz: Sul. Morphia 1-6 gr.; acid arsenious 1-20 gr.; pil. pulv. ext. coloc :c. 2½ grs.; podophylin ¼ gr.; pil. cinchonidia sul. i gr.; pil. monobromated camphor i gr.; pil. phosphorus comp. (phosphorus 1-60 gr. nux vom. ¼ gr.)

I carried the experiment of the B. & C. pills further to determine the quantities of ingredients in each. Of the quinine pills I dissolved several containing five grs. in a quantity of water, acidulated with a few drops of dilute sulphuric acid, from which the quinine was precipitated by water of ammonia, and agitated with ether, which was removed by a pipette to a weighed watch-glass. The quinine was left in a sticky mass after evaporation, which I dried at a moderate temperature, and weighed—thus determining the amount of crystalized sul. quinine. The yield was the full quantity claimed.

The market is filled with spurious coated pills, especially of quinine—since the recent advance in price and great demand; and we should exercise the most scrupulous care to guard against impositions which are being attempted on the profession, as well as the community at large. There is no doubt but the most prominent disadvantage in the use of these pills is their *insolubility*, Some of these nine samples were very difficult to dissolve, only yielding to prolonged application of heat, even after disintegration. Those that did so readily dissolve deserve great praise and credit, and should be remembered by every physician who reads this paper. The most persistent vigilance of the physician, not only as to sugar coated pills, but all pharmaceutical preparations, is the only remedy that will enable us to guard carefully against impositions of this character. The profession of the pharmacist will be yet more advanced and elevated to that perfect standard which is of such vital importance, when the products of the manufacturer come to be more frequently and critically examined. Testimony, at last, is the only way of arriving at the value of any of these pharmaceutical preparations.

[It is gratifying to know that this nice method of administering drugs can be relied upon, at least, in the case of those prepared by certain of our large and well known manufacturers in this line. We can substantiate the statement of our contributor on the above article, having for a number of years used these sugar pills with great satisfaction, especially with delicate and fastidious patients.—ED.

